# Eco-Friendly 3D-Printed Concrete Made with Waste and Organic Artificial Aggregates

**DOI:** 10.3390/ma17133290

**Published:** 2024-07-03

**Authors:** Karolina Butkutė, Vitoldas Vaitkevičius, Fausta Adomaitytė

**Affiliations:** Faculty of Civil Engineering and Architecture, Kaunas University of Technology, Studentų g. 48, 51367 Kaunas, Lithuania; vitoldas.vaitkevicius@ktu.lt (V.V.); fadomaityte@gmail.com (F.A.)

**Keywords:** municipal solid waste bottom slag, hemp shives, charcoal, 3D-printed concrete, artificial aggregate

## Abstract

In this research, the results of an experimental study on the use of three alternative components for creating artificial aggregates (AAs) (granules) and their usage in 3D-printed concrete (3DPC) are examined. This study combines AAs made from organic components like hemp shives (HSs), pyrolyzed coal (charcoal), waste/municipal solid waste incinerator bottom slag (BS), and a mix of a reference 3DPC with the aforementioned AAs. Particularly, to enhance these properties to make low-carbon 3DPC, in this research, the potential of using AAs as lightweight aggregates was increased to 14% in terms of the mass of the concrete. Each mix was tested in terms of its printability via a preliminary test in a 3D printing laboratory. For an additional comparison with the aforementioned cases, 3DPC was mixed with unprocessed hemp shives, charcoal, and BS. Furthermore, their strength was measured at 28 days, and lastly, their durability parameters and shrinkage were experimentally investigated. Cross-sections of the fragments were studied under a scanning electron microscope. In this study, we achieved improvements in the mechanical properties of AAs for their development and implementation as an innovative way to reduce carbon in 3DPC.

## 1. Introduction

In the last few years, it has become increasingly necessary to use sustainable building materials to achieve the 2050 goal of a carbon-neutral building industry. The main initiator of this sustainability plan is the French government, which has already released a law stating that at least 50% of buildings need to be built using timber or other natural materials [1]. Additionally, organic aggregates used in 3DPC include coffee grounds [2], oyster shells [3], and municipal solid waste incineration ash [4]. Three-dimensional printed concrete is composed of rice husk ash, marble dust, and burnt ashes from municipal solid waste incinerators, which are considered sustainable mixing materials [5].

Cogeneration power plants have become more and more popular in the last few decades, by which large amounts of waste and bottom slag are generated; because of this, there has been intensive research on where these plants should be located. The accumulation of BS in landfills can pose challenges for waste management. There is an advantage to incineration in that it recovers energy from the burning process and produces additional heat or energy. The remaining inflammable residue amounts to 5–10% by volume from the municipal solid waste (MSW) stream [6]. Likewise, BS could be used as a replacement in mortar as a recycled fine aggregate or as a part of natural or lightweight coarse aggregates in green concrete. In some ways, granules made from BS could replace all of the natural gravel in concrete and road base layers or be used as a secondary aggregate in asphalt applications [7,8,9].

One of the newest research projects in this field investigates biomaterials, namely mycelium-based composites from the fibrous root-like system of fungi, which brings us one step closer to achieving the use of 50% organic materials in building components in the building industry [10]. And it is not the only suitable solution for 3D-printed concrete using organic materials. Organic waste from agricultural industries used in 3D-printed concrete projects also could include corn cob powder as a thermal insulation material [11]. Despite this, the greatest amount of effort to discover ‘green’ 3D-printed concrete materials is still focused on hemp. It is a building material that is well known for its insulating properties and environmental friendliness. Hemp is an organic material, a biotype of the species *Cannabis sativa* (*C. sativa*), known for its industrial applications in Europe and Asia [12]. The earliest known stories of hemp cultivation date back 5000 years to China; however, industrial hemp production probably dates back even further to ancient Egypt [13]. The most cultivated areas in the world are in countries like Canada, China, France, and the USA [14]. The first hemp-based concrete material used in construction in the 1990s, “hempcrete”, was a bio-based concrete made of hemp shreds or hurds combined with water, sand, and a lime-based binder [15]. Also, because of its high porosity, hemp exhibits hygroscopic activity, making it very susceptible to changes in temperature, liquid water, and vapor. This characteristic reveal how sensitive hemp shiv in hempcrete is to changes in humidity. The morphology of this bio-based material can be significantly impacted by swelling and shrinking [16].

Most organic materials contain a level of sucrose, their primary carbohydrate component. Hemp also has a minimal sucrose level. Sucrose is a common organic compound classified as a disaccharide sugar. Its molecular formula is C12H22O11. To neutralize sucrose, in most cases, aluminium sulphate is used. It does not let sucrose form in organic materials and reduces their hygroscopicity and water absorption [17]. Additionally, the sucrose content of a different organic material will be measured in this study.

One more frequently used organic material in grilling today is charcoal. We are accustomed to charcoal being used only for food preparation, but it is noted that ancient Egyptians turned wood into charcoal in ancient times [18]. In the 16th century, Sweden’s manufacturing industry started to produce charcoal at 400–500 °C [19]. Pyrolysis (slow, flash, and fast) is the chemical decomposition of carbon-based organic material through heat in the absence of oxygen [20,21]. As a result of this, gaseous and liquid products are formed, as well as a solid residue. More importantly, from this green technology, we can obtain green hydrogen, ethylene, propylene, electricity, district heat, wood vinegar, wood tar, and bio-oils [22]. The leading countries in the fast pyrolysis of biomass are Canada, Finland, Germany, the Netherlands, the UK, and the USA. Indeed, charcoal is lightweight, has insulating and absorptive properties, and can enhance concrete porosity, particularly when it is used in lightweight concrete or concrete bricks as a replacement for sand [23,24,25].

## 2. Materials and Methods

### 2.1. Binder Materials

The main binder material used in this study was ordinary Portland cement (OPC) (CEM I 42,5 R, AB “Akmenes cementas”, Naujoji Akmenė (Lithuania)). The other added binder was CL 90-S-class hydrated lime (HL) (Lhoist Bukowa Sp. z o. o., Bukowa (Poland)). As an additional material in the granules and 3D-printed composite compositions, we used burnt oil shale ash–burnt fly ash (BFA) from “Enefit Power AS” (Narva, Estonia). The chemical compositions of these binder materials are listed in Table 1. In all 3DPC composites, we used the same amounts of binders: OPC—30%, HL—2%, and BFA—9%.

### 2.2. Aggregates

As already mentioned, binder materials in the 3DPC compositions are aggregates. We mainly used natural aggregate and sand from UAB “Rizgonys” quarry (Lithuania). It was dried and sieved with a size range of 0–2 mm. The 3DPC compositions had differing amounts of natural aggregate, from 55 to 41%. 

**Figure 1:**
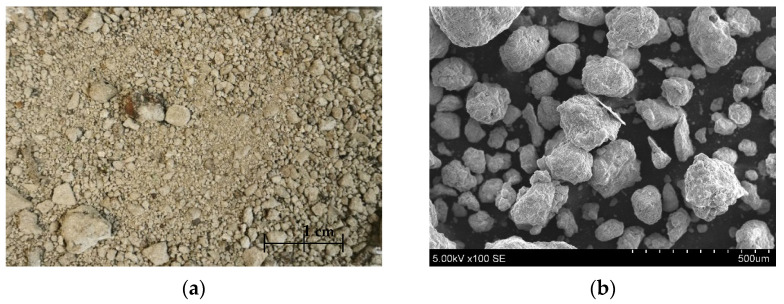
Picture of BS from plant (**a**), SEM of BS (×)100 (**b**).

**Figure 2:**
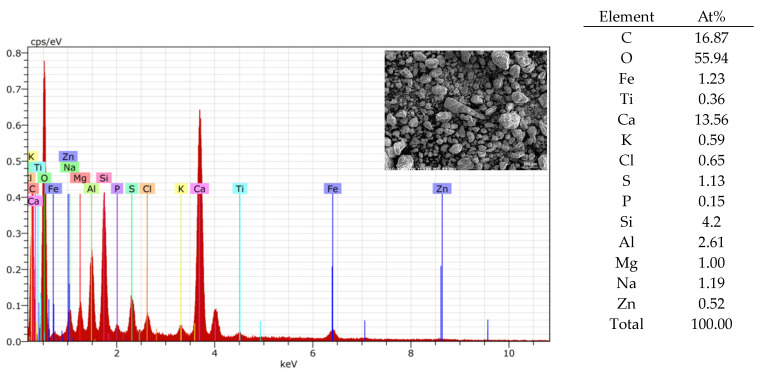
BS spectrum analysis (EDS).

Another non-hazardous waste aggregate material used for artificial aggregate formation was bottom slag (BS), sieved with a 4 mm sieve (UAB “Kauno kogeneracinė jėgainė”, (Kaunas, Lithuania)). Table 2 lists all common chemical compositions of BS. A detailed view of BS is shown in Figure 1. Figure 2 shows its detailed element composition with EDS analysis. Slag usually consists of non-combustible materials and ash residues.

**Figure 3:**
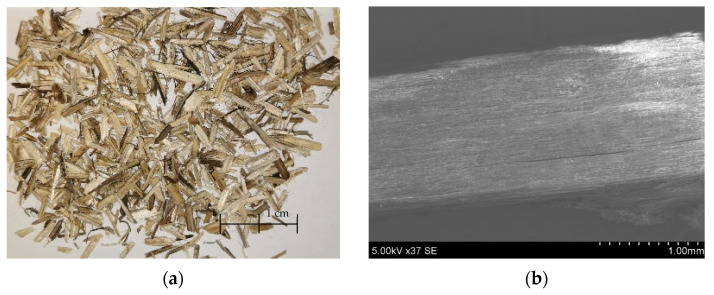
Picture of HSs from plant (**a**), SEM of HSs (×)37 (**b**).

Another organic material was used for making AA: cleaned hemp shives (HSs) (UAB “Natūralus Pluoštas”, Kėdainiai (Natural Fiber), Lithuania) (Figure 3). This organic material before the granulation process was milled in a cutting mill (Pulverisette 15, “Fritsch GmbH”, (Idar-Oberstein, Germany)) to ensure that most of the particles were shorter than 4 mm. 

**Figure 4:**
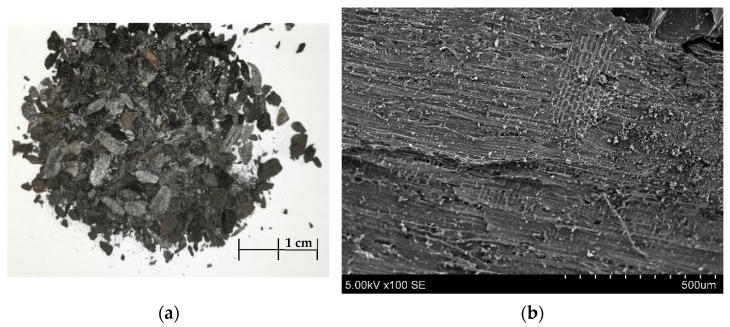
Picture of charcoal after milling (**a**), SEM of charcoal (×)100 (**b**).

Lastly, another organic material used in this research study was pyrolyzed coal, also called charcoal (Figure 4), from a local supplier, made in Ukraine. Before being used to make AA, charcoal was crushed with cutting mill (Pulverisette 15, “Fritsch GmbH” (Idar-Oberstein, Germany)) and sieved with a 4 mm sieve. Charcoal is produced during heating, in which volatile compounds are removed, leaving behind a porous carbon structure. When one burns wood to make charcoal, the additional components, like sucrose, present in the starting material undergo decomposition into its component sugars and ultimately into carbon, water vapor, and other by-products. This is the main conclusion from the analysis of the sucrose measurement results of Table 3. 

### 2.3. Additives

To obtain additional properties in 3DPC, we used the following lateral additives: Calcium formate—an accelerator to control hardening and setting time (“Mudanjiang Fenga Chemicals Imp. & Exp. Corp”, (Mudanjiang, China)); Denka—for the regulation of expansion (“Neuvendis SPA”, (San Vittore Olona, Italy)); Peramin, a sulfonated melamine polymer, which was used as a plasticizer (“Imerys S.A.”, (Paris, France)); Vinnapas hydrophobic dispersive polymer powder was used to achieve adhesion and hydrophobic effect between layers (“Wacker Chemie AG”, (Burghausen, Germany)); and Polypropylene fibre (with a 3 mm length) for reinforcement of and reduction in cracks (“Belgian fibers manufacturing”, (Mouscron, Belgium)). The dry composition of the materials comprised 4% of the total concrete mass.

### 2.4. Test Method and Sucrose Measurement

Many organic materials or plants have sucrose in their sap. It serves as a transportable form of energy and is composed of two simpler sugars, glucose and fructose, which are linked together. Glucose is one of the main animal and plant carbohydrates [26]. 

Even though hemp shives typically do not contain high levels of sucrose, in an alkaline environment (hydrolysis), it is a possibility that alkaline substances (cementitious systems), such as hydroxide ions, could break chemical bonds. For hemp, this might be applied to break down cellulose and hemicellulose into simpler sugars [27].

In additive manufacturing, because of their effectiveness, sugars have been employed for a long time as setting retarders. Sugars have long been employed to delay hydration [28]. If sugars are not properly removed or if they undergo reactions during the concrete curing process, they might potentially affect the properties of the concrete. For example, sucrose and citric acid negatively influence the compressive strength and flexural stress of cementitious system compositions [29]. 

In this investigation, this retarding property should be avoided. In this study, for the measurement of sucrose content in investigated organic components, a sugar refractometer (ATC 0–32% Brix pro), an apparatus that determines a sample’s Brix content (sucrose content) automatically by calculating its refractive index, is used [30]. °Bx (% mass sucrose) is measured using an optical refractometer with a scale between 0 and 5. The amount of sugar in an aqueous solution is measured in degrees Brix (°Bx). One degree Brix, which expresses the strength of the solution as a percentage by mass, is equal to one gram of sucrose in 100 g of solution [31]. See results in Table 3.

### 2.5. Granulation and Preparation of Artificial Aggregate

**Figure 5:**
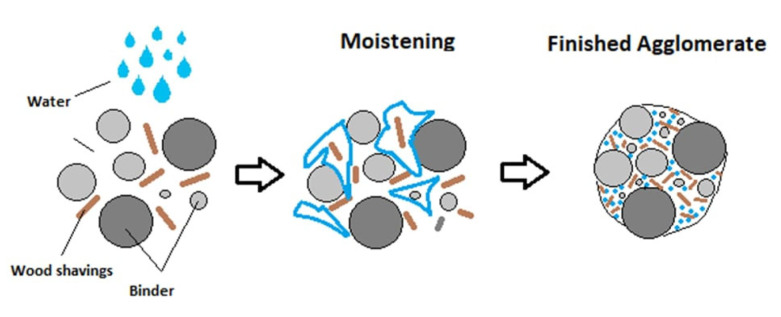
Schematic diagram of AA formation—granulation process [34].

Millions of tons of trash are accumulated annually in landfills worldwide. It is necessary to assess crushing, grinding, and screening methods to alleviate the problem of bottom slag building up in landfills [32]. One of the explored sustainable and environmentally friendly options for managing and reducing the impact of this waste stream is the granulation of BS with binding materials, making lightweight aggregate. After that, it can be used in mortar, concrete, road base layers, or even asphalt applications with a range of different granulometry sizes [7,33]. The granulation process is pictured in Figure 5.

**Figure 6:**
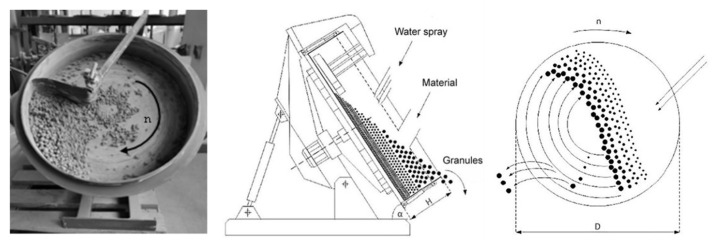
Disk granulator (rotating pan) method [35] and photo.

In contrast to how inorganic materials like OPC or BS are used for building properties, organic materials are not usually used for granulation, aside from the pelletizing procedure, which turns waste wood into uniformly sized little pellets that can be burned as fuel. In the context of granulation, organic materials can present unique challenges and considerations. Biologic materials such as HSs are more likely to decompose, rot, or release odours when exposed to moisture and microbial activity. As a result, their granulation and processing differ from those of inorganic materials. Because of this, the granulation of organic materials must be taken into consideration. For this reason, AA was mechanically produced in Figure 6 using the agitation granulation method. Moistened materials were rotated in a disk granulator during the cold bonding process in the absence of an external compacting force. 

Parameters of the disk granulator were as follows: disk height (H)—100 mm, disk diameter (D)—500 mm, revolution speed (n)—35 rounds per min, inclination angle (α)—45°, and load ratio—0.8–1.4 kg.

HSs’ ability to absorb (approx. 211–317%) and retain water makes them a versatile and valuable material in a variety of industries, especially in sustainable construction [36]. Similarly, charcoal is a highly porous form; it increases a material’s surface area and enhances its ability to absorb a wide range of substances, including gases, liquids, and impurities. Charcoal’s water absorption capacity is especially useful in the production of AA. The water absorption values of the compositions of granules with HSs and charcoal were updated and are listed in the results section (Section 3.1). These materials’ water absorption property is used to absorb other binder materials and make homogenous granules.

For this research, six contrasting designs of artificial aggregate were made (Section 3.1). Following granulation, newly made granules were evenly distributed in a thin layer on a horizontal surface to naturally dry for three days at 20 ± 2 °C and with a relative humidity of 50 ± 5%. Granules were segregated using a 4 mm wire mesh filter to 0–4 mm after drying them. In particular, the granule diameter (4 mm) was chosen following comparison with the natural aggregate diameter already used in reference 3D-printed concrete (composite numbers 0–3), after taking into account our intention to make items and assessing 3D printing laboratory equipment. After being prepared, granules were tested in 3DPC composites (Section 2.7) in fresh state (Section 3.2), and their strength results were compared (Section 3.2).

### 2.6. Tests of Artificial Aggregate

In the laboratory, we defined artificial aggregate type and artificial aggregate particle density. From the artificial aggregate, we selected 10 most-rounded granules, put them into the laboratory heating oven at 105 °C, and dried them until constant weight was reached. Following drying, a tiny layer of wax was applied to each granule, and hydrostatic scales were used to weigh them. Averaged results of different granule particle densities are shown in Section 3.1 [37]. 

The bulk density was measured using a one-litre bowl. While the bowl was raised, the granules were dropped at the speed of free fall.

Energy dispersive spectrum analysis (EDS), a Hitachi S-3400N (“Hitachi High-Technologies Corporation”, (Tokyo, Japan)), and a scanning electron microscope (SEM) were used to investigate the microscopic structure of artificial aggregate and 3DPC elements.

### 2.7. Tests for Fresh State 3D-Printed Concrete

Table 4 lists all analysed composites—3DPC compositions with AA. As a reference for analysis, we mixed composites 0–3.

**Figure 7:**
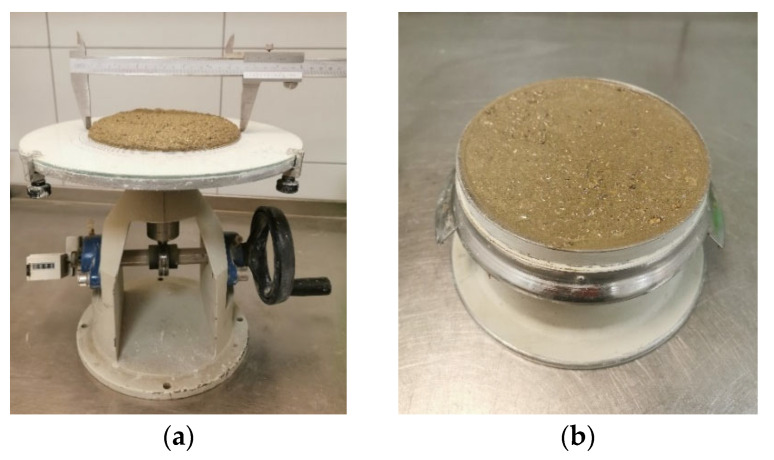
Test equipment for freshly mixed 3D-printed composite: flow table (**a**), 1 litre pot (**b**).

Initially, the flow of newly mixed composites (Table 4) was assessed using the EN 1015-3 standard’s flow table test (Figure 7a) [38]. Following this test, bulk density in a 1 L bowl was determined by EN 1015-6 standard (Figure 7b) [39]. Lastly, prisms (4 × 4 × 16 cm) were produced to measure strength after the freshly mixed composites’ properties were tested.

### 2.8. Test of 3D-Printed Concrete

**Figure 8:**
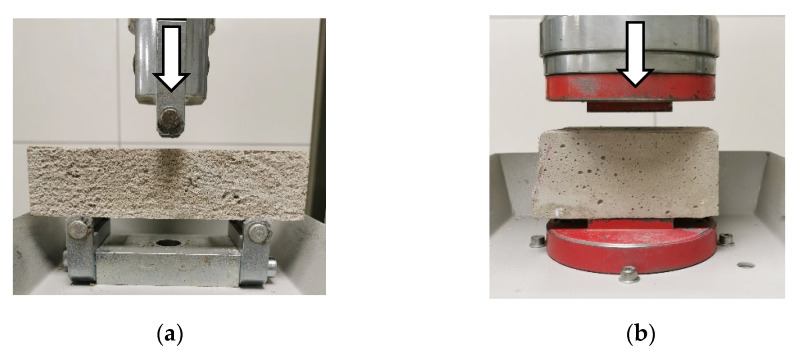
Flexural (**a**) and compressive (**b**) strength tests of 3DPC prisms [42,43].

After mixing the 3DPC composites with water with hand-held mixing drill (Table 4), prisms were made (moulded) according to EN 1015-11 standard [40]. Prisms were cured for 28 days in a laboratory setting at a temperature of 20 ± 2 °C and a relative air humidity of 65 ± 5%, adhering to the aforementioned requirements. Following that, prisms’ flexural and compressive strengths were determined using a compression and bending testing machine (referred to as the “ratio TEC”). The load without shock at a uniform rate of 10 N/s was applied. Both measuring description methods are shown in Figure 8 [41]. Results are listed in (Section 3.2).

**Figure 9:**
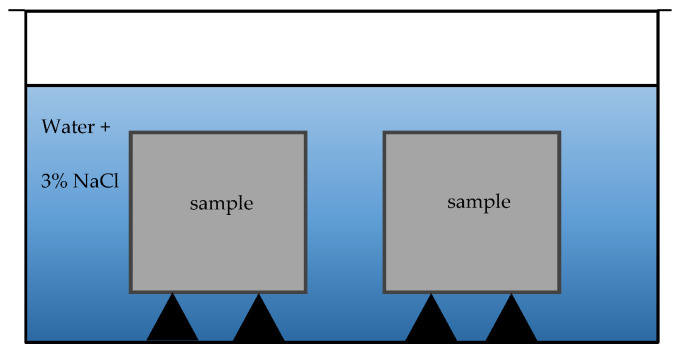
Free–thaw resistance test method for samples.

For freeze–thaw resistance, laboratory test was performed using methodology from CEN/TS 12390-9 standard [44]. Frost resistance was accomplished for sample prisms that were 4 × 4 × 16 cm (Figure 9). In total, samples were tested for 25 and 100 cycles.

To test deformation, 3DPC was moulded into 4 × 4 × 16 cm prisms. After 1 day, prism ends were glued to special retainers for measuring changing dimensions with a monometer (Mitutoyo”, (Kawasaki, Japan)). For 28-day period, prisms were held at 20 ± 2 °C and a relative humidity of 50 ± 5%.

### 2.9. CO_2_ Calculation

Organic materials are used in 3DPC for a reason—to reduce carbon emissions. Organic materials like trees and wood absorb carbon dioxide, which is why biogenic carbon ($P_{{CO}_{2}}$) content can be determined by the formula in EN 16449 standard [45].

### 2.10. Trial 3D Concrete Printings

**Figure 10:**
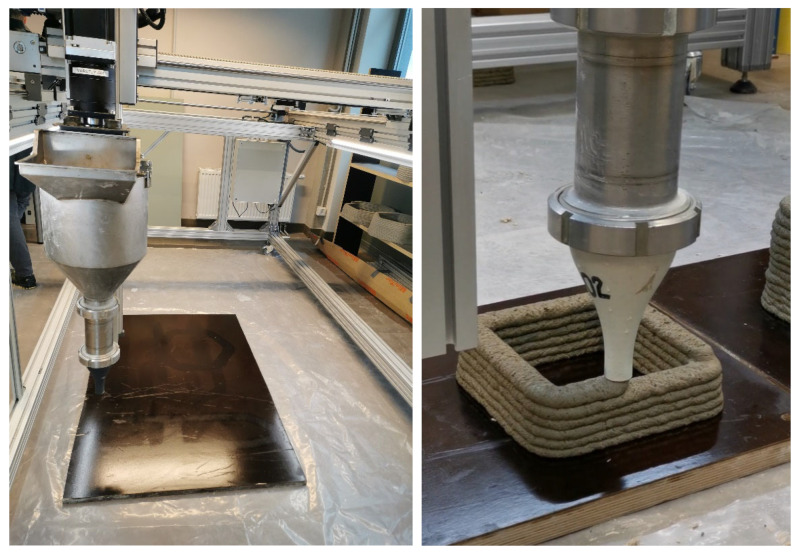
3DPC construction and demonstration of sample printing.

For 3D printing, Riga Technical University (RTU) designed a special gantry-style printer suitable for making construction components like concrete. With an aluminium frame measuring 2000 × 1000 × 1200 (h) mm, the printer can accommodate models up to 1500 × 1000 × 1000 mm and has a 30 L hopper volume. Open-source Repetier-Firmware was used to manage the printer, while Simplify3D 5.1 software from Simplify 3D Ltd. (Cincinnati, OH, USA) was used for slicing. Three-dimensional models were made with the Solidworks 3D CAD program, Waltham, JAV. The dimensions of the printer head nozzle were 20 mm in diameter, 35 to 45 mm in layer width, and 10 mm in layer height. Figure 10 displays the printer’s design.

## 3. Results

### 3.1. Artificial Aggregate Results

The AA compositions are listed in Table 5.

**Figure 11:**
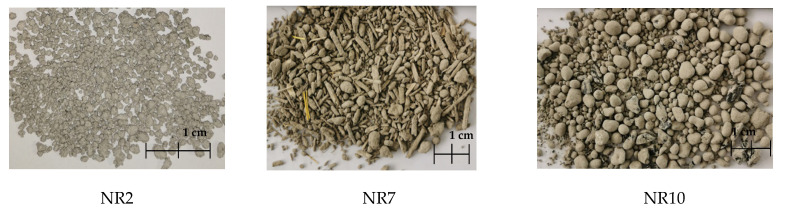
Examples of artificial aggregate granules that were made.

The visual results after the granulation process are shown in Figure 11.

**Figure 12:**
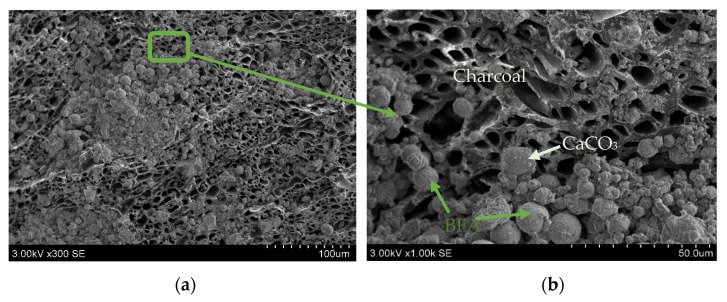
Resulting artificial aggregate NR9 surface, (×)300 (**a**); and more detailed SEM photo (×)1000 (**b**)**.**

The absorption capacity of activated charcoal is a crucial feature that contributes to its effectiveness in 3D concrete printing applications. It is important to note that activated charcoal is used for specific purposes, and the type and quality of its absorption can vary, so it is essential to take an SEM photo to investigate the contact zone for the intended application (Figure 12). As expected, Figure 12 shows promising results for the contact zone of the charcoal and binder materials after the granulation process. It is seen that AA could be made to be very solid. Furthermore, an SEM analysis was selected to understand the microstructure of the materials, the connection between the binder materials and organic materials, how these components interact, and whether they create a homogenous system. The connection between the granules and compositions gives us more information about the strength results. 

**Figure 13:**
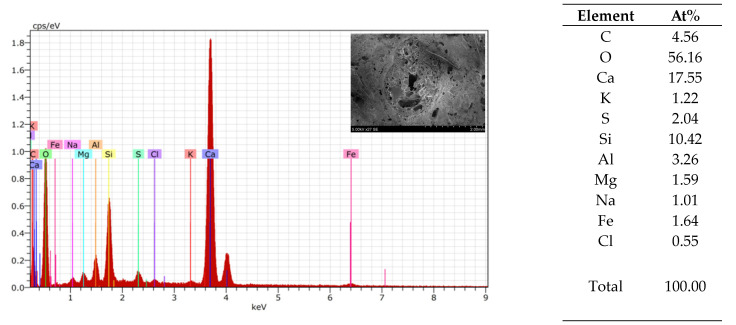
Granule EDS of mix 1–6.

**Figure 14:**
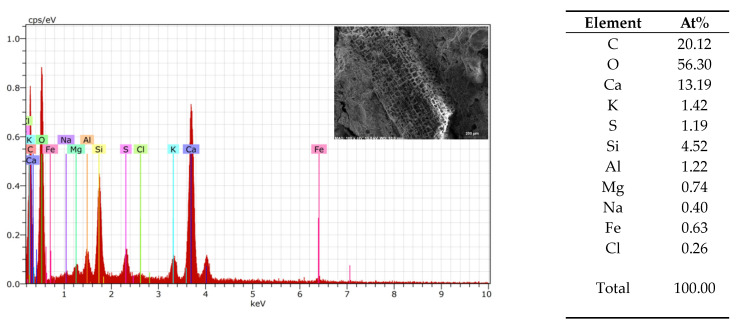
Granule EDS of mix 1–9

**Figure 15:**
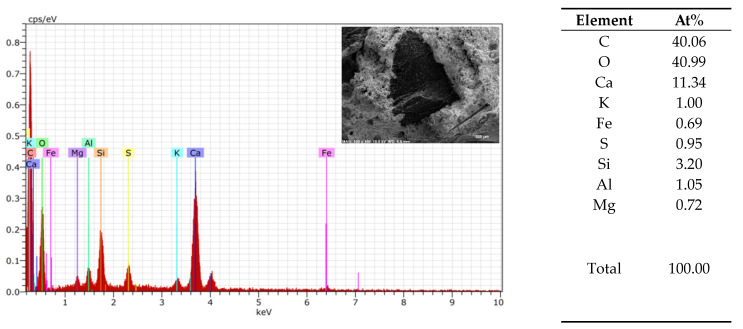
Granule EDS of mix 1–11.

An EDS analysis was used to compare the similarity of the 3DPC of each tested material in the AAs (Figure 13, Figure 14 and Figure 15). From the element analysis, it could be observed that the carbon amount significantly increases when there are more organic components in the analysed area

In Table 6 are listed averaged results of different granule particle densities.

### 3.2. 3D-Printed Concrete Results

Based on the analytical technique Nazmul et al. suggested, the literature shows dosage rates of 1–3 percent for hemp shives [46]. This case study was experimental, and as many as hemp shives possible were poured into the 3D-printed concrete composition to keep the concrete’s printability. Our results show the same percentage.

In Table 7, the freshly made 3DPC shows one of the main parameters for printability—consistency. The printability test analysis showed that the optimal consistency for printing (specifically with the 3D printer in the RTU laboratory) is 16–17 cm. For an evaluation of the largest possible load of granules in the 3DPC, one experimental batch was printed using a 50% composition of granules in place of the aggregate. The consistency parameter showed a 17.3 cm flow. In summary of this printing experiment, it could be stated that half of the aggregate variation presents positive results for future research, as listed in 1–8 results.

Flexural and compressive strengths results of hand-made prisms after 28 days are listed in Table 8.

**Figure 16:**
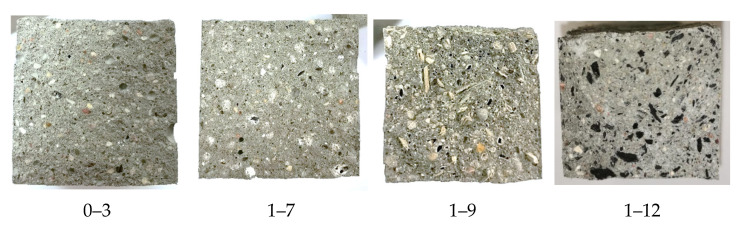
Prism crosscuts after 28 days.

After the strength tests, we took photos of crosscuts that were the most characteristic for each composition (Figure 16).

In all of the crosscuts (Figure 16), we noticed that the granules’ arrangement in the prisms had an approximately even distribution. 

When comparing all three additives (BS, HSs, and charcoal), a difference was noted between the granulated and non-granulated forms in the 3DPC. The HSs and charcoal behave differently when they are in granulated form and require more water. In particular, the HSs showed a level of immersion that was three times greater than that of the granulated HSs in the 3DPC.

The workability of the 3DPC with AA and with HSs is found to be worse than that of the 3DPC with loose HSs. The concrete becomes almost unprintable. In addition, all their compressive strength results are reduced. A small increase in flexural strength might show that in the concrete, the HSs work as a fibre but not as an aggregate.

In contrast, non-granulated HSs and BS were added to the 3D-printed concrete, and their workability and strength in freshly made concrete after 28 days were compared. One of the impact factors of adding non-granulated BS is sieving; it needs to be sieved using the maximum particle size diameter of the concrete aggregate—in this specific case, 4 mm.

Considering the results of the milled charcoal (granulated and loose), we noticed that the increase in the amount of water shows a greater immersion of the charcoal particles in water, which leads to a deterioration in all its fresh and hardened parameters. Despite this, a composition could be successfully printed with the granulated charcoal.

**Figure 17:**
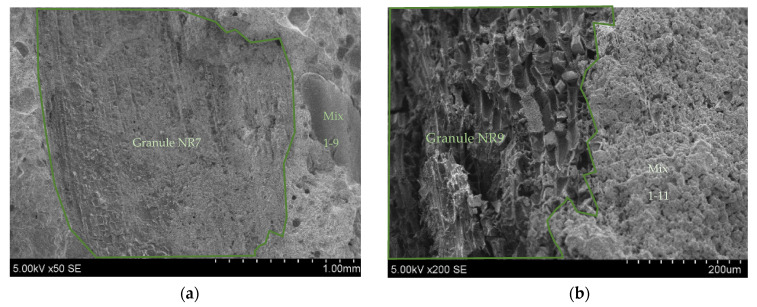
Prism crosscuts with granules in mix 1–9 (**a**) and mix 1–11 (**b**).

Figure 17 shows the SEM images of both of the analysed 3DPC mixes, for which there is a strong connection between the granules and hardened composition mass.

**Figure 18:**
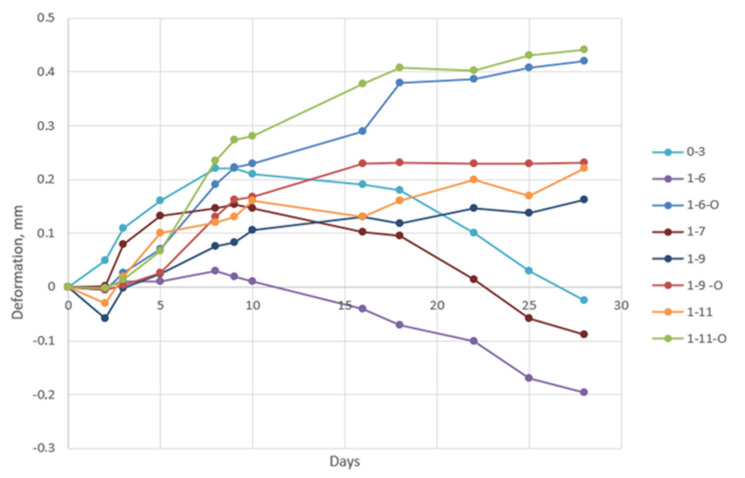
Deformation graph for first 28 days.

When comparing the deformation results in Figure 18, we noticed that all the compositions with loose waste/organic materials showed worse results than the composition prisms with AA. An assumption is made about this phenomenon: using AA as an aggregates is more advantageous than using 3DPC without granulation. The deformation results of mixes 1–7 and 1–9 showed positive results in terms of the stability of the system.

To evaluate the impact of different temperatures, we conducted a freeze–thaw resistance test based on the methodology of the CEN/TS 12390-9 standard [47]. The results are listed in Table 9.

After 25 cycles of the freeze–thaw resistance test, the samples with the non-granulated organic materials started to crack and lost small parts from their surface. However, the other samples with the granulated materials successfully underwent 100 cycles without any cracks or fallout.

### 3.3. CO_2_ Reduction

Table 10 and Table 11 list the possible amount of carbon dioxide emissions taken into the investigated 3DPC composites.

To analyse their carbonation advantage, AA granules of NR2 and NR3 were put into a carbonization chamber for 2 days. The density was measured before and after their carbonation. The results are listed in Table 10. As shown, the granules absorbed 160–222 kg/m^3^ of CO_2_ [48]. One of the most innovative carbon emission capture and mineralization companies, “Carbon8”, is researching the possibility of the uptake of 10–28% CO_2_ by granule weight [49]. Moreover, the CO_2_ concentration in the flue, which is between 40 and 100% after 20 min, has the same amount of CO_2_ uptake. This shows that the carbonization process takes place stably despite the continuous flow of smoke through the flue.

The calculations were performed according to the EN 16449 standard, and the results (Table 11) showed that 3DPC mixes 1–9 and 1–10 with AA NR7 and NR8 (with HSs) have the same ability to absorb CO_2_, because they have the same amounts of organic components.

The NR9 and NR10 AA granules are composed of 19.5% charcoal by mass. The 3DPC composites (mixes 1–9 and 1–10) have about 10.5% charcoal. Charcoal has a large surface area and acts like a kind of hardened sponge [50]. A total of 1 g of activated carbon (charcoal) could adsorb about 13.2 g of CO_2_ [51]. Researchers have also determined that 1 kg of concrete with 30% charcoal by weight could remove roughly 13 g of CO_2_ [52,53]. Summarizing these facts, it could be concluded that theoretically, the aforementioned compositions could absorb about 4.3 kg/m^3^ of CO_2_ from the concrete by mass. However, detailed future studies are needed to know the true values.

## 4. Conclusions

Research on the granulation process indicates that organic materials like HSs could be beneficial in 3DPC and need to be kept safe from the negative effects of wet environments. Approximately 80% of the total mass of the granules (with a >4 mm diameter) that are acceptable for printing can be obtained with a slow water spray and a rotation speed of 35 rounds per minute. 

Comparing the AAs’ SEM images, we noticed that both organic materials firmly absorb the moist and dry mass of the binders, forming spherical or round granules. In comparison with charcoal, HS AA becomes more brittle after sieving, and the bond between the HSs and the binder layer decreases.

In terms of strength results, granulated and loose BS is unambiguously favourable. In contrast, granules with burnt oil shale ash and lime have slightly weaker results. Mix 1–7 display nearly the same results as the reference and are indisputably appropriate for the 3D printing of items. This indicates the benefit of granulation for materials with matching dimensions of particles.

The deformation results of the reference mix were worse than those of the 3DPC compositions 1–7 and 1–9 with granules from BS and HSs. Presumably, the BS is sufficiently stable, and the HSs act like a fibre in the composite to achieve balance. Moreover, all the granulated organic components make the 3DPC more stable with smaller deformations compared to those of 3DPC with non-granulated organic components. The expansion regulator regulates deformations in composites only when the organic components are granulated and slows their reactivity when the organic materials are not granulated, according to the deformation graphic.

The samples without the granulated organic components had inferior accession results (merely 2.2–2.7%) compared to those of the same materials in the AA when their freeze–thaw resistance levels were evaluated. Conversely, the non-granulated BS achieved accession results that were 9.5% better than the results of the AA with BS.

In summary, the use of HS AA alongside non-granulated BS is an excellent way to reduce CO_2_ in 3DPC.

## Figures and Tables

**Table 1:** Chemical compositions of binder materials.

Material	SiO_2_ (%)	Al_2_O_3_ (%)	Fe_2_O_3_ (%)	CaO (%)	MgO (%)	SO_3_ (%)	Na_2_O (%)	K_2_O (%)	Cl (%)	CO_2_ (%)	Density kg/m^3^
CEM I 42.5 (OPC)	18–20	4–5	3–4	62–65	3–4	3.3	0.1	1–1.5	-	-	3150
Hydrated lime (HL)	-	-	-	94–96	0.3–0.4	0.05–0.10	-	-	-	0.5–4.0	2240
Burnt fly ash (BFA)	27	7	4–5	45–51	4–5	9	0.15	3–4	0.47	-	2640

**Table 2:** Chemical compositions of BS.

Material	SiO_2_ (%)	Al_2_O_3_ (%)	Fe_2_O_3_ (%)	CaO (%)	MgO (%)	SO_3_ (%)	Na_2_O (%)	K_2_O (%)	Cl (%)	TiO_2_ (%)	Mn_2_O_3_ (%)	P_2_O_5_ (%)	Density kg/m^3^
Bottom slag (BS)	46.11	7.66	9.46	20.14	2.65	3.71	3.01	1.27	0.89	1.17	0.14	2.11	2550

**Table 3:** Sucrose measurement results.

	Charcoal	Hemp Shives	Birch Sawdust	Conifer Sawdust
Sugar content, % (°Bx)	0	0.2	0.8	1.9

**Table 4:** 3DPC designs.

Materials	Name of 3DPC and Amount of Materials in kg/m^3^										
	0–3	1–6	1–6-O	1–7	1–8	1–9	1–9-O	1–10	1–11	1–11-O	1–12
CEM I 42,5 R	339	356	340	357	377	369	347	369	369	378	369
Sand 0–2 mm	523	410	392	412	292	425	507	425	426	436	426
Calcium hydroxide	13	14	14	14	15	15	14	15	15	15	15
Burnt shale ash	85	90	86	90	95	93	87	93	93	95	93
Additives	40	42	40	42	44	43	41	43	43	44	43
Bottom slag (0–4 mm)	-	-	129	-	-	-	-	-	-	-	-
Hemp shives (0–4 mm)	-	-	-	-	-	-	4	-	-	-	-
Milled charcoal (0–4 mm)	-	-	-	-	-	-	-	-	-	31	-
Granules NR2	-	88	-	-	-	-	-	-	-	-	-
Granules NR3	-	-	-	85	177	-	-	-	-	-	-
Granules NR7	-	-	-	-	-	56	-	-	-	-	-
Granules NR8	-	-	-	-	-	-	-	56	-	-	-
Granules NR9	-	-	-	-	-	-	-	-	54	-	-
Granules NR10	-	-	-	-	-	-	-	-	-	-	54

**Table 5:** Artificial aggregate granule compositions.

Materials, g	Granules Number					
	NR2	NR3	NR7	NR8	NR9	NR10
Bottom slag	1000	1000	-	-	-	-
Burnt oil (shale) ash	-	300	540	540	540	540
Portland cement CEM I 42.5	-	100	182	120	182	120
Calcium hydroxide	400	-	-	60	-	60
Hemp shives	-	-	50	50	-	-
Charcoal	-	-	-	-	175	175
Water	300	300	185	185	120	135

**Table 6:** AA measured density.

	Artificial Aggregate Name					
	NR2	NR3	NR7	NR8	NR9	NR10
Particle density, kg/m^3^	1448	1450	1016	1022	981	982
Bulk density, kg/m^3^	1091	1109	623	649	785	718

**Table 7:** Freshly made 3DPC’s parameters.

3DPC Name	Water, L/kg	Flow, cm	Bulk Density, kg/m^3^
0–3	0.145	16.4	2100
1–6	0.150	16.6	2030
1–6-O	0.195	18.0	2000
1–7	0.154	17.7	2060
1-8	0.164	17.3	2010
1–9	0.183	16.0	1940
1–9-O	0.235	14.6	1800
1–10	0.200	16.1	1930
1–11	0.200	17.4	1950
1–11-O	0.220	16.0	1570
1–12	0.193	15.8	1910

**Table 8:** Prism strength results after 28 days.

Composite Number	Prisms of 40 × 40 × 160 mm		
	Density, kg/m^3^	Flexural Strength, MPa	Compressive Strength, MPa
0–3	2027	6.9	44.1
1–6	1949	5.3	40.8
1–6-O	1914	5.9	43.0
1–7	1926	6.2	43.3
1-8	1900	4.2	36.3
1–9	1891	5.0	37.9
1–9-O	1730	5.9	24.9
1–10	1895	3.9	34.7
1–11	1875	4.7	35.3
1–11-O	1457	4.0	21.2
1–12	1859	3.7	32.7

**Table 9:** Freeze–thaw resistance test impact on strength results.

Composite Number	Compressive Strength Result before Cycles	Compressive Strength Result before 25 Cycles	Escalation, %	Compressive Strength Result before 100 Cycles
0–3	42.76	46.10	+7.8	57.18
1–6	45.23	47.78	+5.6	59.56
1–6-O	33.73	38.83	+15.1	-
1–7	40.17	42.15	+4.9	49.26
1–9	24.34	29.39	+20.7	34.21
1–9-O	16.81	19.92	+18.5	-
1–11	21.86	25.16	+15.1	31.13
1–11-O	15.21	17.10	+12.4	-

**Table 10:** CO_2_ emissions of BS AA.

	AA Name	
	NR2	NR3
Density, not carbonated kg/m^3^	1448	1450
Density, carbonated, kg/m^3^	1670	1610
CO_2_ absorbed, kg/m^3^	222	160
Uptake, %	15.3	11.0
Reduced CO_2_ amount in 1 m^3^ of 3DPC, kg/m^3^	28.5 (mix 1–6)	20.2 (mix 1–7)

**Table 11:** CO_2_ emissions of HS AA.

	AA Name	
	NR7	NR8
Density, kg/m^3^	1016	1022
Amount of HSs in 1m^3^ of 3DPC, %	4.5	4.5
Reduced CO_2_ amount in 1 m^3^ of 3DPC, kg/m^3^	29 (mix 1–9)	29 (mix 1–10)

## Data Availability

Data of this study are available from the corresponding author upon reasonable request.
